# Cigarette smoking may accelerate the progression of IgA nephropathy

**DOI:** 10.1186/s12882-021-02453-4

**Published:** 2021-06-29

**Authors:** Siqing Wang, Aiya Qin, Gaiqin Pei, Zheng Jiang, Lingqiu Dong, Jiaxing Tan, Li Tan, Yi Tang, Wei Qin

**Affiliations:** 1 West China School of Medicine, Sichuan University, Chengdu, Sichuan China; 2 Division of Nephrology, Department of Medicine, West China Hospital of Sichuan University, Guoxue Alley 37, Chengdu, 610041 Sichuan China; 3 Division of Rehabilitation, Department of Medicine, West China Hospital of Sichuan University, Chengdu, China; 4 Key Laboratory of Rehabilitation Medicine in Sichuan Province, Chengdu, Sichuan China

**Keywords:** IgA nephropathy, Cigarette smoking, Propensity score matching, Renal survival

## Abstract

**Background:**

Whether cigarette smoking is associated with the progression of immunoglobulin A nephropathy (IgAN) remains uncertain; therefore, we aimed to evaluate the effect of cigarette smoking on the prognosis of IgAN.

**Methods:**

We divided 1239 IgAN patients from West China Hospital of Sichuan University who met the inclusion criteria into smoker (current or former) and non-smoker groups. The endpoint was end-stage renal disease (ESRD: eGFR < 15 mL/min/1.73 m^2^ or undergoing renal replacement treatment) and/or eGFR decreased by > 50%. Kaplan–Meier, correlation, logistic regression and Cox proportional hazards analyses were performed. The association between cigarette smoking and IgAN was further verified by propensity-score-matched cohort analysis.

**Results:**

During the mean follow-up period of 61 months, 19% (40/209) of the smoker group and 11% (110/1030) of the non-smoker group reached the study endpoint (*p* < 0.001). Multivariate Cox regression analysis revealed that cigarette smoking (hazard ratio (HR) = 1.58; *p* = 0.043) was an independent risk factor predicting poor renal progression in IgAN, and that IgAN patients with chronic kidney disease (CKD) stage 3–4 were more susceptible to cigarette smoking (*p* < 0.001). After propensity score matching (PSM), a significant correlation between cigarette smoking and renal outcomes in IgAN patients was seen. Furthermore, Spearman’s correlation test revealed that smoking dose was negatively correlated with eGFR (*r* = 0.141; *p* < 0.001) and positively related with proteinuria (*r* = 0.096; *p* = 0.001).

**Conclusions:**

Cigarette smoking is an independent risk factor for IgAN progression, especially for advanced patients.

## Background

Immunoglobulin A (IgA) nephropathy (IgAN) is the most common primary glomerulonephritis and a leading cause of end-stage renal disease (ESRD) [1]. IgAN is diagnosed by renal biopsy, which is characterized by the deposition of IgA immune complexes and 20–40% of IgAN patients reach ESRD 10–20 years after the initial diagnosis [1]. As a result, it is of great importance to identify the risk factors for IgAN to delay progression to ESRD. The prevalence of cigarette smoking is increasing worldwide, especially in China [2], and smoking is a risk factor among chronic kidney disease (CKD) patients [3, 4]. A case–control study revealed that smoking contributed to the progression to chronic renal failure, especially regarding nephrosclerosis and glomerulonephritis [4]. However, few studies have analyzed the relationship between cigarette smoking and IgAN, and results are controversial [5, 6]. Additionally, renal pathologic parameters were not considered in all patients, in previous studies. Therefore, the aim of this study was to investigate whether cigarette smoking has an effect on the progression of IgAN patients.

## Methods

### Study design

The diagnosis of IgAN was based on renal biopsy, which showed a predominance of IgA deposits in the glomerular mesangium, either alone or with IgG, IgM, or complement C3 [7]. We recruited patients with renal biopsy-confirmed IgAN from West China Hospital of Sichuan University between January 2009 and December 2018. The inclusion criteria were as follows: (1) > 18 years old; (2) renal biopsy- confirmed IgAN; and (3) followed-up for at least 1 year before reaching the endpoint of our study. The exclusion criteria were as follows: (1) systemic diseases (including but not limited to systemic lupus erythematosus, diabetes mellitus, Henoch–Schönlein purpura, liver cirrhosis); (2) presence of ESRD; and (3) insufficient pathologic data (renal biopsies with < 8 glomeruli) or missing follow-up data. The study was approved by the Ethical Committee of West China Hospital of Sichuan University (FF-33-2019), and written informed consent for participation in the study was obtained from all patients.

### Clinical and pathological data

We divided the patients into non-smoker and smoker (current or former) groups. Smoker was defined as having actively smoked > 400 cigarettes in a patient’s lifetime [8]. The patients’ demographics and baseline clinical data were collected at the time of renal biopsy and constituted sex, age, serum creatinine, blood pressure, and 24-h urine protein. Estimated glomerular filtration rate (eGFR) was calculated using the CKD epidemiology collaboration (CKD-EPI) equation. Hypertension was defined as blood pressure > 140/90 mmHg or using antihypertensive agents. To study the relationship between the amount of cigarette smoking and the progression to our study endpoint in IgAN patients, we used the special definition, pack-years, which was calculated as the number of cigarettes per day multiplied by the number of years of smoking, divided by 20 [9]. We then studied categories of pack-years (0, 1–10, 11–20, and > 20 pack-years) according to the quartiles of all patients’ pack-years. Renal pathology changes were reviewed by experienced pathologists and nephrologists in accordance with the Oxford classification: mesangial hypercellularity (M0/M1); endocapillary hypercellularity (E0/E1); segmental glomerulosclerosis (S0/S1); tubular atrophy/interstitial fibrosis (T0/T1/T2); and cellular or fibrocellular crescents (C0/C1/C2) (METS-C) [10].

### Endpoint

The endpoint was ESRD, which was defined as eGFR < 15 mL/min/1.73 m^2^ or requiring renal replacement treatment, and/or eGFR decreased by > 50% compared with the time of renal biopsy.

### Statistical analysis

All statistical analyses were performed using IBM SPSS statistical software (version 26.0; IBM Corp., Armonk, NY, USA). Categorical data were analyzed using Chi-square tests and were presented as frequencies (percentages). Continuous variables were expressed as mean ± standard deviation (SD) and were analyzed with analysis of variance, Kruskal–Wallis H test, Student’s t-test, or nonparametric Mann–Whitney U test. Kaplan–Meier (K–M) survival analysis and Cox regression models were also performed. Results were expressed as hazard ratios (HR) and 95% confidence intervals (CI). To control the significant differences in demographic and clinicopathological characteristics between smokers and non-smokers, we performed propensity-score matching (PSM) according to important clinical and pathologic factors (sex, hypertension, blood pressure, 24-h urine protein, serum creatinine, eGFR, treatment, and Oxford MEST-C score). Smokers were matched to non-smokers with 1:3 nearest neighbor matching without replacement (the caliper width was set at 0.2) to address the marked differences between the groups [11]. Statistical significance was considered at *p* < 0.05.

## Results

### Demographic and clinicopathological characteristics

This study involved 1588 patients; 349 patients were excluded because of secondary IgAN (*n* = 31), eGFR < 15 mL/min/1.73 m^2^ (*n* = 2), insufficient pathologic data (*n* = 277), and insufficient follow-up period (*n* = 39). Finally, 1239 patients met the inclusion criteria (Table 1). The mean follow-up time was 60.8 ± 28.7 months, and the patients’ mean age (at the time of renal biopsy) of smokers compared with nonsmokers was 38.6 ± 11.5 years vs 33.2 ± 10.8 years, respectively. The proportion of male patients was much higher among smokers, and hypertension was reported in 37.8% of the smokers. Before PSM, there were significant differences between the two groups for sex, blood pressure, 24-h urine protein, serum creatinine, and pathologic lesions of tubular atrophy/interstitial fibrosis.

**Table 1:** Demographic and clinicopathological characteristics of 1239 IgAN patients and 497 patients matched by propensity score

Characteristics	Before PSM		After PSM	
	Non-smokers (1030)	Smokers (209)	Non-smokers (318)	Smokers (179)
Clinical				
Male (%)	351 (34.1)	203 (97.1) ^**^	299 (94)	173 (96.6)
Hypertension (%)	252 (24.5)	79 (37.8) ^**^	98 (30.8)	64 (35.8)
SBP (mmHg)	127.0 ± 18.2	127.6 ± 16.5	129.2 ± 17.4	128.2 ± 16.2
DBP (mmHg)	83.0 ± 13.2	83.0 ± 13.4	84.9 ± 12.8	83.7 ± 13.1
Serum creatinine (umol/L)	88.3 ± 41.3	116.6 ± 54.2 ^**^	108.5 ± 49.3	115.8 ± 55.3
eGFR (mL/min per 1.73m^2^)	93.8 ± 31.3	82.8 ± 33.7 ^**^	90.7 ± 32.3	85.0 ± 34.3
Urine protein (g/24 h)	2.1 ± 2.5	2.7 ± 3.0 ^**^	2.3 ± 2.7	2.4 ± 2.6
Treatment				
Support treatment (%)	436 (42.3)	77 (36.8)	145 (45.6)	68 (38)
Prednisone or other immunosuppressive agents (%)	594 (57.7)	132 (63.2)	173 (54.4)	111 (62)
Pathologic	Oxford Classification			
M1 (%)	774 (75.2)	116 (77)	243 (76.4)	137 (76.5)
E1 (%)	47 (4.6)	12 (5.7)	9 (2.8)	9 (5)
S1 (%)	623 (60.5)	130 (62.2)	189 (59.4)	115 (64.2)
T1/T2 (%)	179 (17.4)	58 (27.8) ^**^	79 (24.8)	51 (28.5)
C1/C2 (%)	239 (23.2)	49 (23.4)	77 (24.2)	38 (21.2)

**Note**: Values for categorical variables are given as number (percentage); values for continuous variables are given as mean ± standard deviation

*stands for *p* < 0.05, ** stands for *p* < 0.01

***Abbreviations***: *SBP* Systolic blood pressure, *DBP* Diastolic blood pressure, *eGFR* Estimated glomerular filtration rate, *M* Mesangial proliferation, *E* Endocapillary proliferation, *S* Segmental sclerosis, *T* Tubular atrophy/interstitial fibrosis, *C* Crescents, *PSM* Propensity-score matching method, *IgAN* Immunoglobulin A nephropathy

### Relationship of cigarette smoking and smoking dose with clinical and pathologic parameters

The correlation analyses indicated that smoking was correlated with tubular atrophy/interstitial fibrosis lesions (odds ratio (OR) = 1.826; 95% CI: 1.296–2.573; *p* = 0.001) but not with other pathological lesions. Moreover, smoking dose was negatively correlated with eGFR (*r* = 0.141; *p* < 0.001) and positively correlated with proteinuria (*r* = 0.096; *p* = 0.001). There was a significant negative correlation between smoking status and sex (Kendall’s tau-b = − 0.475; *P* < 0.001), indicating that smoking was more popular with men than women, in our patient cohorts.

### Cigarette smoking was an independent risk factor for IgAN patients

Univariate cox regression analysis results revealed that cigarette smoking, hypertension, sex, serum creatinine, 24-h urine protein, mesangial hypercellularity, segmental glomerulosclerosis, and tubular atrophy/interstitial fibrosis were significantly associated with renal outcomes. After adjusting for all of the important factors, multivariate Cox regression analysis showed that cigarette smoking remained an independent risk factor for IgAN progression (Table 2).

**Table 2:** Cox proportional hazard model for the renal outcome in 1239 IgAN patients

Parameters	Univariate	Multivariate
	HR (95%CI)	HR (95%CI)
Smoker	1.97 (1.37–2.83) ^**^	1.58 (1.02–2.46) ^*^
Female	0.64 (0.47–0.89) ^**^	2.00 (1.29–3.10) ^**^
Hypertension	3.22 (2.32–4.45) ^**^	1.50 (1.04–2.16) ^*^
SBP	1.03 (1.02–1.03) ^**^	
DBP	1.04 (1.03–1.05) ^**^	
Serum creatinine	1.02 (1.01–1.02) ^**^	1.01 (1.09–1.01) ^**^
eGFR	0.96 (0.96–0.97) ^**^	
Urine protein	1.10 (1.06–1.15) ^**^	1.04 (0.97–1.10)
Oxford Classification		
M1	2.38 (1.41–4.01) ^**^	1.61 (0.95–2.73)
E1	1.22 (0.62–2.40)	0.63 (0.31–1.31)
S1	2.97 (2.03–4.37) ^**^	1.59 (1.06–2.39) ^*^
T1/T2	7.65 (5.51–10.60) ^**^	3.13 (2.09–4.67) ^**^
C1/C2	1.30 (0.92–1.84)	1.07 (0.74–1.55)

**Note**: * stands for *p* < 0.05, ** stands for *p* < 0.01

***Abbreviations***: *HR* Hazard ratio, *95% CI* 95% confidence interval, *SBP* Systolic blood pressure, *DBP* Diastolic blood pressure, *eGFR* Estimated glomerular filtration rate, *M* Mesangial proliferation, *E* Endocapillary proliferation, *S* Segmental sclerosis, *T* Tubular atrophy/interstitial fibrosis, *C* Crescents, *IgAN* Immunoglobulin A nephropathy

### Relationship between the amount of cigarette smoking and renal survival

**Fig. 1:**
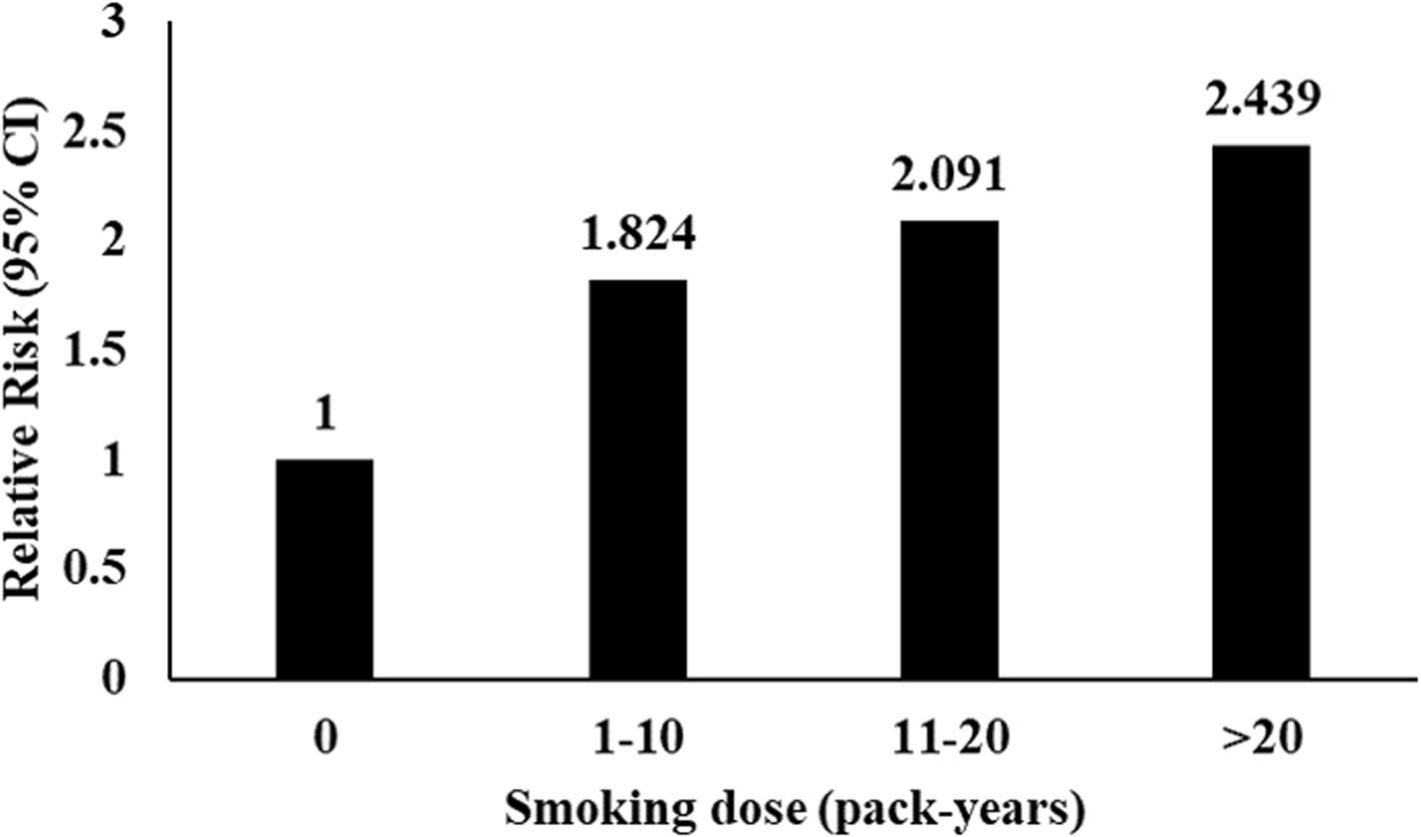
Relative risk for endpoint associated with categories of smoking dose

Patients were divided into four groups according to the pack-years quartiles (0, 1–10, 11–20, and > 20 pack-years) to evaluate the dose-related association between smoking and renal outcomes in IgAN patients. Our results revealed that smoking dose effected renal outcome and pathologic structural changes in IgAN patients. Smokers with > 20 pack-years tended to have higher proportions of tubular atrophy/interstitial fibrosis. The logistic regression analysis revealed that smokers with > 20 pack-years had an increased risk of developing our study endpoint of 143% compared with non-smokers, and a dose-response relationship was found for this risk factor (Fig. 1).

### Effect of cigarette smoking on renal outcomes

**Fig. 2:**
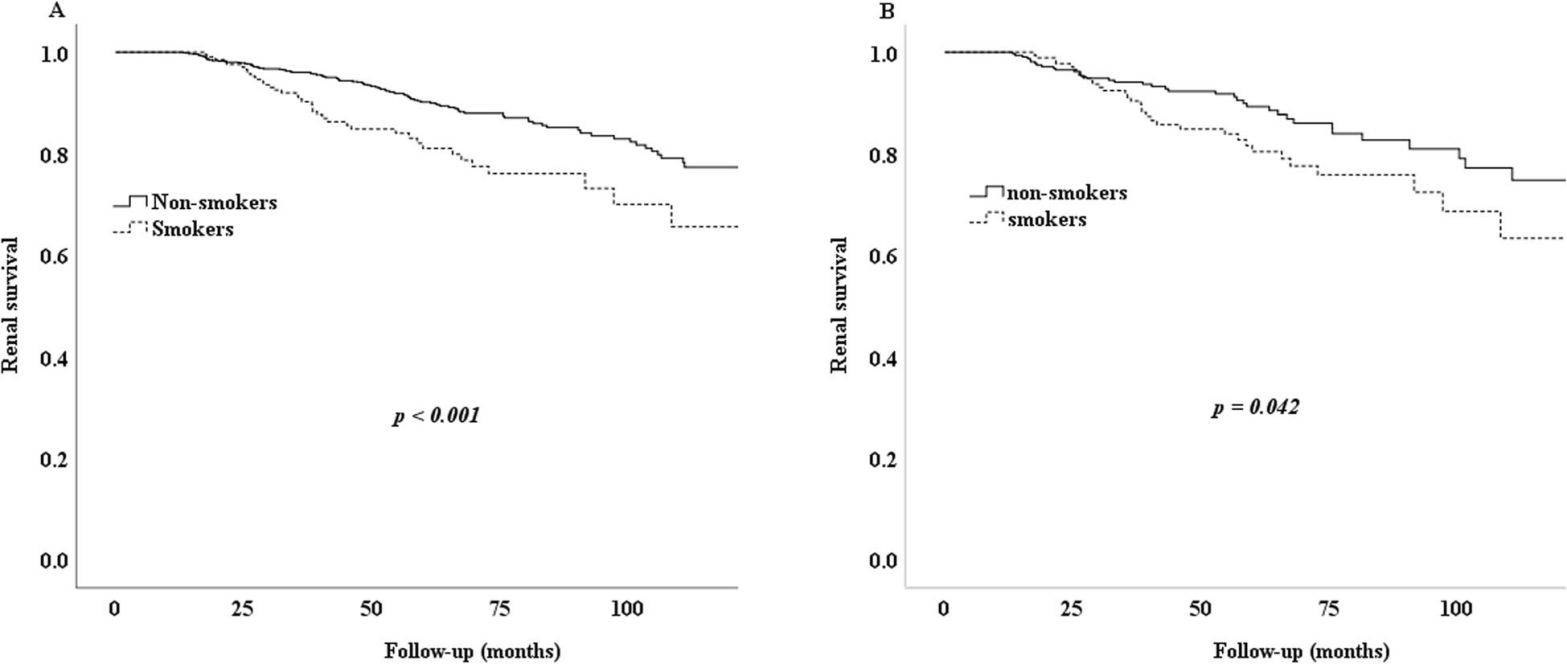
Kaplan-Meier analysis for the endpoint of cigarette smoking. Note: **A** Kidney survival rates in non-smoker and smoker group; **B** Kidney survival rates in non-smoker and smoker group matched by propensity score

**Fig. 3:**
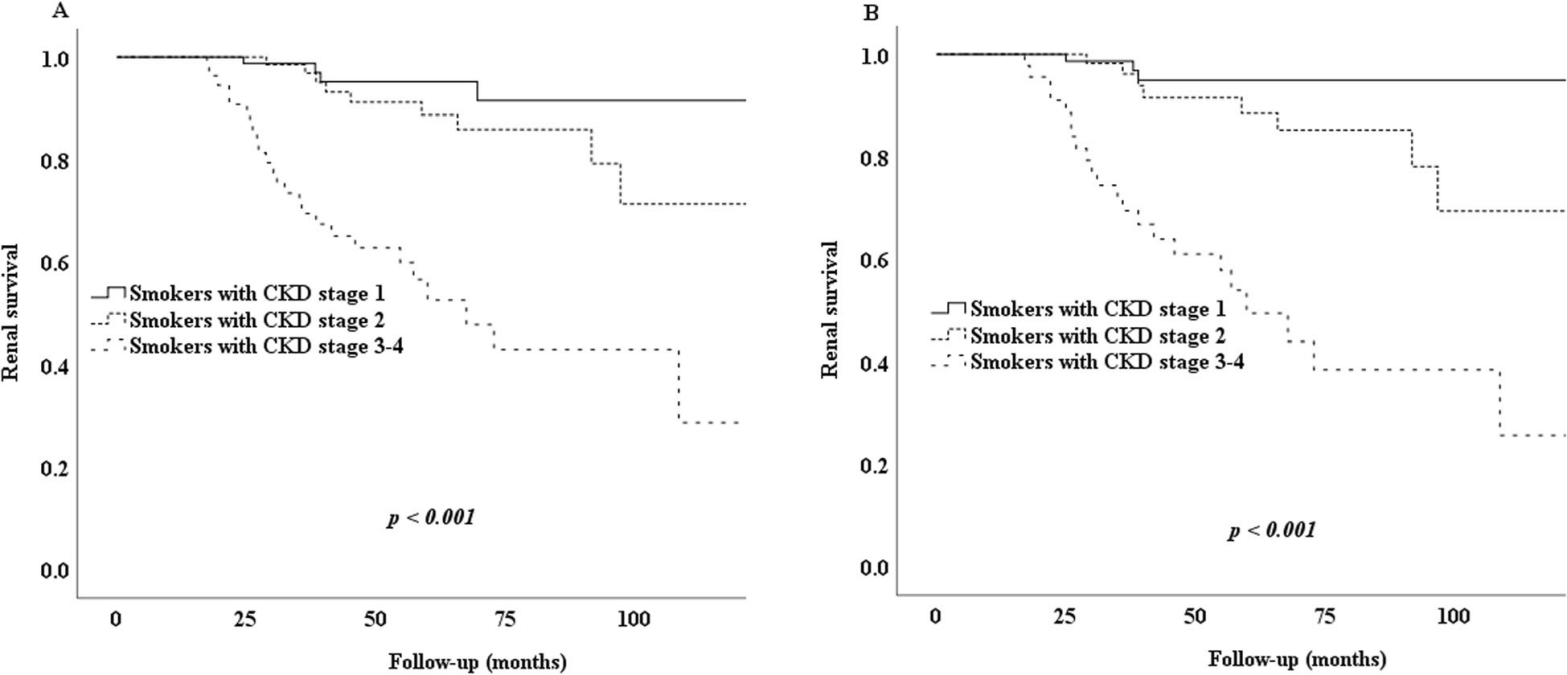
Kaplan-Meier analysis for the endpoint of smoker patients with different CKD stages. Note: **A** Kidney survival rates in smoker patients with stage 1 or 2 or 3–4 CKD group; **B** Kidney survival rates in smoker patients matched by propensity score with stage 1 or 2 or 3–4 CKD group

K–M survival analysis (before or after PSM) indicated that cigarette smoking was related to poor renal survival in IgAN patients. Many more smokers (19%, 40/209) than nonsmokers (11%, 110/1030) reached the study endpoint in the non-PSM cohort (*p* < 0.001) (Fig. 2A). We also found that 13% (40/318) of non-smokers and 20% (35/179) of smokers reached the endpoint in the PSM cohort (*p* = 0.042) (Fig. 2B). Further analysis showed that patients with more severe renal dysfunction were more susceptible to the effects of cigarette smoking (Fig. 3). We classified patients into three groups (CKD stage 1, CKD stage 2, CKD stage 3–4) according to their eGFR. Before PSM, renal survival rates in the three groups differed significantly according to the K–M curves (*p* < 0.001) (Fig. 3A). The PSM cohort analysis confirmed the result (Fig. 3B). Before PSM, the logistic regression analysis showed that smokers with CKD stage 2 and stage 3–4 had increased risks of developing our endpoint of 224 and 1670%, respectively, comparing with CKD stage 1 smokers.

### Hypertension and renal vasculopathy were associated with cigarette smoking and IgAN progression

**Fig. 4:**
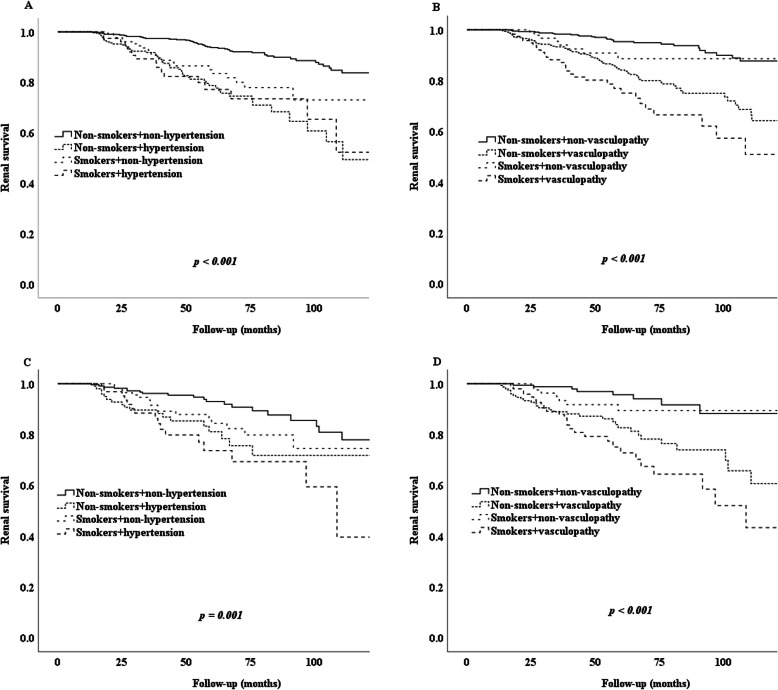
Kaplan-Meier analysis for the endpoint of cigarette smoking, hypertension and renal vasculopathy. Note: **A** Kidney survival rates in non-smoker and smoker group in patients with hypertension and without; **B** Kidney survival rates in non-smoker and smoker group in patients with renal vasculopathy and without; **C** Kidney survival rates in non-smoker and smoker group matched by propensity score in patients with hypertension and without; **D** Kidney survival rates in non-smoker and smoker group matched by propensity score in patients with renal vasculopathy and without

Compared with non-smokers, smokers had a higher risk of hypertension (OR = 1.876; 95% CI: 1.371–2.567; *p* < 0.001) and renal vasculopathy (OR = 1.569; 95% CI: 1.163–2.118; *p* = 0.003). Further analysis indicated that smokers with hypertension and renal vasculopathy had the worst renal outcomes (before or after PSM), indicating that cigarette smoking, hypertension, and renal vasculopathy could accelerate IgAN progression (Fig. 4).

## Discussion

IgAN is the primary glomerulonephritis with the highest incidence in the world [7], and approximately 20% of patients will progress to ESRD within 20 years after diagnosis [1]. The deleterious effects of smoking have been investigated in CKD patients; current smoking was an independent risk factor for progression of microalbuminuria, macroalbuminuria, and ESRD among diabetic nephropathy patients [12]. A retrospective study of IgAN showed that cigarette smoking was an important predictor of IgAN progression [5]. In contrast, another observational study found that cigarette smoking had no direct connection with IgAN [6]. However, renal pathological changes were not evaluated in these studies. Moreover, confounding factors between smoking and non-smoking participants were not adjusted using PSM. Therefore, the association between cigarette smoking and IgAN prognosis remains unclear.

In the current study, 1239 patients with IgAN were followed-up for 61 months, and we revealed that cigarette smoking was significantly associated with renal survival in these patients. During the follow-up, a significantly higher portion of smokers (19%) reached the study endpoint of our study compared with non-smokers (11%). Moreover, smokers had an increased rate of our study endpoint of 98%.

Further analysis indicated that cigarette smoking was related to more severe tubular atrophy/interstitial fibrosis. Smoking could induce oxidative stress and increase the stiffness of central vessels, causing tubular damage and increasing the risk of tubular atrophy and fibrosis [13, 14]. Additionally, pathologic structural changes in tubular atrophy/interstitial fibrosis are a risk predictor of renal outcomes for IgAN patients [15]. In our study, there was a dose-response relationship between cigarette smoking and renal outcomes. Smokers with > 20 pack-years had an increased risk of progressing to our study endpoint of 143% compared with non-smokers. Clinically, smoking dose was positively correlated to 24-h urine protein concentration and negatively correlated to eGFR. This may explain why smokers experienced dismal renal outcomes.

Patients with more severe renal dysfunction were more susceptible to the effects of cigarette smoking. The logistic regression analysis showed that smokers with stage 2 and stage 3–4 CKD had increased risks of progressing to our study endpoint of 224 and 1670%, respectively. It may be that patients with severe renal dysfunction have a decreased ability to resist the effects of smoking, which may involve poor renal reserve, poor residual renal function, poor anti-inflammatory response, and an anti-fibrosis effect. Higher smoking doses are associated with higher toxins, more severe renal damage, and a worse prognosis. According to our results, we suggest that IgAN patients with stage 3 or 4 CKD should undergo smoking cessation therapy to slow deterioration of the renal disease.

In our study, IgAN patients who were smokers were more likely to have hypertension and renal vasculopathy changes, and poorer renal outcomes compared with patients without hypertension who did not smoke. Smoking may theoretically cause renal injury through the pathogenic effects of nicotine [16, 17]. The direct harmful effects on the vasculature promoting renal atherosclerosis are a possible major mechanism [14]. It has also been suggested that nicotine may promote the proliferation of mesangial cells and affect endothelial function [16, 17]. The pathological feature of IgAN is diffuse mesangial cell proliferation and mesangial matrix increase [16, 17], and our analyses showed that cigarette smokers with worse hypertension and renal vasculopathy changes on biopsy experienced faster IgAN progression and poorer renal outcomes. Thrombotic microangiopathy (TMA) lesions are a proven risk factor for IgAN [18]. Unfortunately, the rate of TMA in our initial cohort was very low; only a few patients had typical renal TMA lesions, and owing to loss to follow-up, these patients were not included in our final analysis cohort. In a future study, we will recruit more IgAN patients and include a longer follow-up time to evaluate the relationships between smoking, TMA, and renal outcomes in IgAN.

To decrease the obvious unbalanced influence of the data, we performed PSM. From the analyses of the matched pairs, we concluded that cigarette smoking is an independent risk factor for IgAN progression. Moreover, the relationships between cigarette smoking and hypertension and renal vasculopathy changes on biopsy were verified by the analyses of the matched pairs. However, our study still has three main limitations. First, this was a retrospective study in a single hospital. Second, the mean follow-up time of 61 months was relatively short, especially for IgAN, which has a very slow progression. Third, the vast majority of smokers in our study were men. Larger studies are needed to clarify whether sex is a risk factor for progression and a worse prognosis in IgAN patients, especially in smokers.

## Conclusions

Cigarette smoking is a significant risk factor for IgAN progression. We must pay more attention to IgAN patients who smoke and who have severe renal dysfunction.

## Data Availability

The datasets used and/or analyzed during the current study are available from the corresponding author on reasonable request.

## Abbreviations

PSM: Propensity score matching
CI: Confidence interval
CKD: Chronic kidney disease
eGFR: Estimated glomerular filtration rate
ESRD: End stage renal disease
IgAN: Immunoglobulin A nephropathy
OR: Odds ratio
HR: Hazard ratio
